# FAM64A Potentiates Bladder Carcinoma Tumorigenesis and Metastasis Through PI3K/mTORC2/AKT Pathway Activation

**DOI:** 10.3390/cancers18030540

**Published:** 2026-02-06

**Authors:** Tao Zhu, Cen Liufu, Cong Yin, Jinqing He, Junhua Luo, Bentao Shi, Yan Wang

**Affiliations:** 1 Department of Urology, Peking University Shenzhen Hospital, Institute of Urology, Shenzhen Peking University-Hong Kong University of Science and Technology Medical Center, Shenzhen 518036, China; 23tzhu@stu.edu.cn (T.Z.); 18fcliu@stu.edu.cn (C.L.); jinqinghz@163.com (J.H.); frank101ljh@163.com (J.L.); 2 Department of Urology, PKU-Shenzhen Clinical Institute of Shantou University Medical College, Shenzhen 518036, China; 3 Department of Urology, Shenzhen Second People’s Hospital, The First Affiliated Hospital of Shenzhen University, Shenzhen 518035, China; congyin0416@163.com; 4 Shenzhen Clinical Research Center for Urology and Nephrology, Peking University Shenzhen Hospital, Shenzhen 518036, China; 5 Central Laboratory, Shenzhen Qianhai Taikang Hospital, No. 3099, Menghai Avenue, Nanshan District, Shenzhen 518000, China

**Keywords:** bladder cancer, FAM64A, PI3K/mTORC2/AKT, EMT

## Abstract

**Background:** FAM64A is highly expressed in various cancers (e.g., breast cancer, ovarian cancer), indicating that it promotes tumorigenesis and progression by facilitating epithelial–mesenchymal transition. In the genitourinary system, dihydrotestosterone promotes the expression of FAM64A by binding of the androgen receptor to the FAM64A promoter, thereby enhancing the proliferation, migration, and cell cycle progression of androgen-dependent prostate cancer cell lines. However, its specific role in the initiation and progression of bladder cancer remains unclear. FAM64A overexpression has been observed in cancers such as breast and prostate; however, its role in bladder cancer (BLCA) is less understood. Muscle-invasive BLCA (MIBC) has a poor prognosis, with five-year survival rates below 50%. This study explores FAM64A’s molecular mechanisms and therapeutic potential in BLCA. **Methods:** FAM64A expression was analyzed using TCGA data and clinical BLCA tissues. Functional assays (CCK-8, wound-healing, Transwell) assessed proliferation, migration, and invasion following FAM64A modulation. Western blotting was used to evaluate EMT markers (Vimentin, Slug) and proteins involved in the PI3K/AKT pathway. Bioinformatics (TCGA/GTEx) identified FAM64A-correlated genes, followed by KEGG pathway analysis. Taselisib (PI3K/AKT inhibitor) validated pathway involvement. **Results:** FAM64A was upregulated in BLCA and correlated with advanced tumor stage, T-stage, and grade. Knockdown suppressed proliferation, migration, and invasion, while overexpression exacerbated these effects. FAM64A promoted G2/M progression (via Cyclin B1/Ki67) and EMT (via Vimentin/Slug). KEGG analysis linked FAM64A to the PI3K/mTORC2/AKT signaling pathway. Taselisib reversed FAM64A-induced EMT and malignant phenotypes. **Conclusions:** FAM64A drives BLCA progression via PI3K/mTORC2/AKT-mediated EMT, serving as a potential prognostic biomarker and therapeutic target for metastatic BLCA.

## Simple Summary

This study establishes FAM64A as a critical driver of bladder cancer progression. We demonstrate that FAM64A accelerates cell cycle progression and induces epithelial–mesenchymal transition (EMT) through activation of the PI3K/mTORC2/AKT signaling pathway, thereby promoting tumor growth and metastasis. The PI3K inhibitor Taselisib effectively reverses these malignant phenotypes induced by FAM64A. These findings nominate FAM64A as both a prognostic biomarker and a promising therapeutic target in bladder cancer.

## 1. Introduction

Bladder cancer represents a significant global health burden within genitourinary malignancies, accounting for >400,000 annual diagnoses worldwide [1,2]. The disease spectrum encompasses non-muscle-invasive (NMIBC) and muscle-invasive (MIBC) subtypes [3,4,5]. NMIBC exhibits high recurrence rates but slow progression [6,7], while MIBC exhibits aggressive metastatic behavior and poor survival outcomes, with 5-year survival rates under 50% [8]. Although over 70% of patients present with NMIBC initially [9,10], the high propensity for progression [11] and recurrence [12] poses significant clinical challenges. Current therapies, including chemotherapy and surgery, have limitations [13], often entailing substantial costs, prolonged treatment, vigilant recurrence monitoring, reduced quality of life, and considerable socioeconomic burden [14]. Therapeutic options for MIBC and metastatic disease remain limited, underscoring the critical need for novel molecular targets.

The FAM64A gene, situated on chromosome 17p13 and spanning eight exons, is also referred to as RCS1, PIMREG, or CATS. Its encoded protein was initially characterized as an interactor of the CALM protein [15]. This molecule is functionally linked to proliferative regulation, displaying elevated expression in leukemias, lymphomas, and multiple tumor types, but minimal detection in quiescent cells [16]. Its high expression in embryonic mouse cardiomyocytes suggests an essential role in proliferation [17] and potential as a cardiac regenerative target via cell cycle promotion [18]. FAM64A also regulates Th17 differentiation and inflammation-associated carcinogenesis [19]. Dysregulated FAM64A expression is observed in multiple cancers, including breast [20], gastric [21], head and neck squamous Cancer [22], and prostate cancer [23], yet its function in BLCA remains unexplored.

The phosphatidylinositol 3-kinase (PI3K)/AKT axis is a central signaling pathway that regulates cellular functions and metabolism and is frequently activated in human malignancies [24]. Substantial evidence confirms the activation of the PI3K/AKT pathway in BLCA, promoting proliferation, metastasis, and survival [25,26]. This cascade maintains oncogenic phenotypes and directly/indirectly induces epithelial–mesenchymal transition (EMT) via pathway crosstalk, facilitating tumor invasion and metastasis [27,28]. Epithelial–mesenchymal transition (EMT) is a process in which epithelial cells lose their apical-basal polarity and cell–cell adhesion, acquire mesenchymal cell phenotypes, and gain migratory and invasive capabilities, thereby promoting metastasis and drug resistance [29]. Numerous studies have demonstrated that EMT is prevalent in bladder cancer and plays a crucial role in regulating drug resistance and muscle invasion/metastasis in urothelial carcinoma [30,31,32]. Consequently, PI3K/AKT pathway inhibition emerges as a promising therapeutic approach for BLCA [33,34,35].

This study demonstrates increased FAM64A expression in BLCA specimens and cell models, which is significantly correlated with adverse clinical outcomes. FAM64A knockdown suppresses malignant phenotypes in vitro and in vivo, while its overexpression exacerbates them. The oncogenic effects of FAM64A, including tumorigenesis and metastasis in BLCA, are driven through the activation of the PI3K/mTORC2/AKT-mediated EMT program.

## 2. Materials and Methods

### 2.1. Gene Databases

TCGA data (https://portal.gdc.cancer.gov/, 21 January 2022) and GEO dataset GSE13507 (https://www.ncbi.nlm.nih.gov/, 23 March 2022) were downloaded to analyze mRNA expression in BLCA versus normal bladder tissues and correlate FAM64A expression with clinicopathological features.

### 2.2. Immunohistochemistry (IHC) for FAM64A

Clinical Specimens: Between January and December 2016, a total of 40 paired tissue samples (BLCA and adjacent normal tissue) were prospectively collected from patients undergoing radical cystectomy at Peking University Shenzhen Hospital. Patients received no prior radiotherapy or chemotherapy. Pathological diagnosis and staging (AJCC TNM 7th edition) were confirmed. Ethical approval (No. 20090017) and informed consent were obtained.

IHC Protocol: Following dewaxing and rehydration, antigen retrieval was performed on the tissue microarray slides (HProA150CS01) with citrate buffer at pH 6.0. Following endogenous peroxidase quenching and serum blocking, the sections were incubated overnight at 4 °C with a rabbit anti-FAM64A primary antibody (Thermo Fisher; Wilmington, DE, USA; 1:20 dilution). Subsequent detection was performed using a biotinylated secondary antibody (ZSGB-BIO, Beijing, China) and DAB for visualization. Counterstaining was performed with hematoxylin.

### 2.3. Cell Culture

Human bladder cell lines—SV-HUC-1 (normal) and BLCA lines (TCC-SUP, 5637, T24, UMUC3, SW780, J82)—were maintained at 37 °C/5% CO_2_ in DMEM (TCC-SUP, UMUC3, T24) or RPMI-1640 (5637, SW780, J82). All media contained 10% FBS, 100 U/mL penicillin-streptomycin, and 1× GlutaMAX.

### 2.4. Cell Transfection

BLCA cells were transfected with FAM64A-specific siRNA (siR-FAM64A) or negative control siRNA (siR-NC) using Lipofectamine 3000 (Invitrogen, Carlsbad, CA, USA). In our experimental setup, two independent siR-FAM64A constructs were utilized and subjected to multiple rounds of validation to ensure consistency in siRNA activity. One construct was selected for all subsequent functional verification studies.

Sequences: siR-FAM64A sense: 5′-AGAAAUUGCUCUUGAACAGGG-3′

antisense: 5′-CUGUUCAAGAGCAAUUUCUGC-3′

siR-NC sense: 5′-UUCUCCGAACGUGUCACGUTT-3′

antisense: 5′-ACGUGACACGUUCGGAGAATT-3′

Lentiviral Transduction for Stable Cell Lines: The FAM64A coding sequence was amplified, cloned into the pLVX-IRES-NEO vector (using EcoRI/NotI sites), sequence-verified, and packaged into lentivirus using the Lenti-X HTX system in 293T cells. T24 and TCC-SUP cells were infected with pLVX-IRES-NEO-FAM64A or empty vector control virus. FAM64A overexpression was confirmed before functional assays.

### 2.5. Western Blotting

Protein lysates were prepared in RIPA buffer supplemented with protease and phosphatase inhibitors. The proteins were resolved on 7.5–10% SDS-polyacrylamide gels, subjected to electrophoresis, and transferred onto PVDF membranes. The membranes were then probed with antibodies against: FAM64A (Thermo Fisher H00054478-D01P, 1:1000), β-tubulin (Proteintech 2013, 1:10,000), Cyclin B1 (CST 12231, 1:1000), Ki-67 (Proteintech 241499E7, 1:10,000), AKT (CST 11E7, 1:1000), mTOR (CST 2983, 1:1000), PI3K (CST 3011, 1:1000), phospho-PI3K (MCE HY-P81211, 1:1000), phospho-AKT-Ser473 (CST 4060, 1:1000), phospho-AKT-Thr308 (CST 13038, 1:1000), phospho-mTOR-Ser2448 (CST 5536, 1:1000), phospho-mTOR-Ser2481 (CST 2974, 1:1000), c-Myc (Proteintech 3D9C12, 1:5000), Vimentin (Proteintech 6K21, 1:20,000), Slug (Proteintech 250290A2, 1:10,000), anti-rabbit IgG-HRP (CST 7074, 1:2000), anti-mouse IgG-HRP (CST 7076, 1:2000).

### 2.6. RT-qPCR

Following extraction with Trizol reagent, total RNA was reverse-transcribed to synthesize cDNA with the PrimeScript RT kit (Takara). FAM64A expression quantified via SYBR Green chemistry (LightCycler 480 II, Germany) with GAPDH normalization. Primers:

FAM64A-F: 5′-TTCTCGGTGGCAGAACATGG-3′

FAM64A-R: 5′-CAGACAGGGCTTGTTTCCTCT-3′

GAPDH-F: 5′-CCACTCCTCCACCTTTGACG-3′

GAPDH-R: 5′-CTGGTGGTCCAGGGGTCTTA-3′

### 2.7. Functional Assays

CCK-8 Proliferation: 3000 cells/well (96-well plate) were assessed at 24, 48, and 72 h. A 10 μL CCK-8 solution (Yeasen) in 100 μL of serum-free medium was added. After 2 h incubation (37 °C, dark), absorbance (450 nm) was measured.

Wound Healing: Confluent monolayers scratched with a 200 μL tip; migration documented at 0/16/24 h.

Transwell Invasion: 3 × 10^4^ cells in serum-free medium seeded onto Matrigel-coated inserts; invaded cells stained with 0.1% crystal violet after 24 h.

### 2.8. Cell Cycle Analysis

sg-NC and sg-FAM64A BLCA cells were subjected to thymidine-mediated synchronization (30 h). Following release, samples were collected at 6–22 h intervals, then fixed, stained with PI (BD Biosciences), and analyzed by flow cytometry (BD Accuri C6 Plus).

### 2.9. Transcriptomic Profiling

RNA sequencing (Novogene, Beijing, China) was performed on FAM64A-overexpressing and CRISPR/Cas9-knockout cells using Trizol (Sangon). DEGs were identified, followed by GO and KEGG enrichment analysis.

### 2.10. Subcutaneous Xenograft Model

T24 cells stably expressing sg-FAM64A or sg-NC control (5 × 10^7^ cells/mL in saline) were mixed 1:1 with Matrigel (BD) and subcutaneously injected into mice (100 μL/mouse). Tumor dimensions were measured weekly; volume = 0.5 × length × width^2^. Mice were sacrificed at 4 weeks for endpoint analysis. Tissues were fixed (10% formalin) for analysis.

### 2.11. Statistical Analysis

Data analysis was performed using IBM SPSS Statistics (v19.0; IBM Corp.) and GraphPad Prism (v10; GraphPad Software). The χ^2^ test examined categorical associations between FAM64A protein expression and clinicopathological characteristics. Survival probabilities were estimated through Kaplan–Meier methodology with log-rank testing. Quantitative data are reported as mean ± standard deviation (SD), obtained from a minimum of three independent replicates. Inter-group comparisons were statistically evaluated using Student’s *t*-test for two-group analyses and one-way ANOVA with Tukey’s post hoc testing for three or more groups. Experiments were performed in triplicate; data expressed as mean ± SD. *p* < 0.05 was considered statistically significant (* *p* < 0.05, ** *p* < 0.01, *** *p* < 0.001).

### 2.12. Bioinformatics Analysis

The limma R package (v4.0) identified DEGs between sgFAM64A and sgNC cells. GO enrichment (BP, MF, CC) and KEGG pathway analyses were performed.

## 3. Results

### 3.1. FAM64A Overexpression in Bladder Cancer Predicts Adverse Prognosis

**Figure 1:**
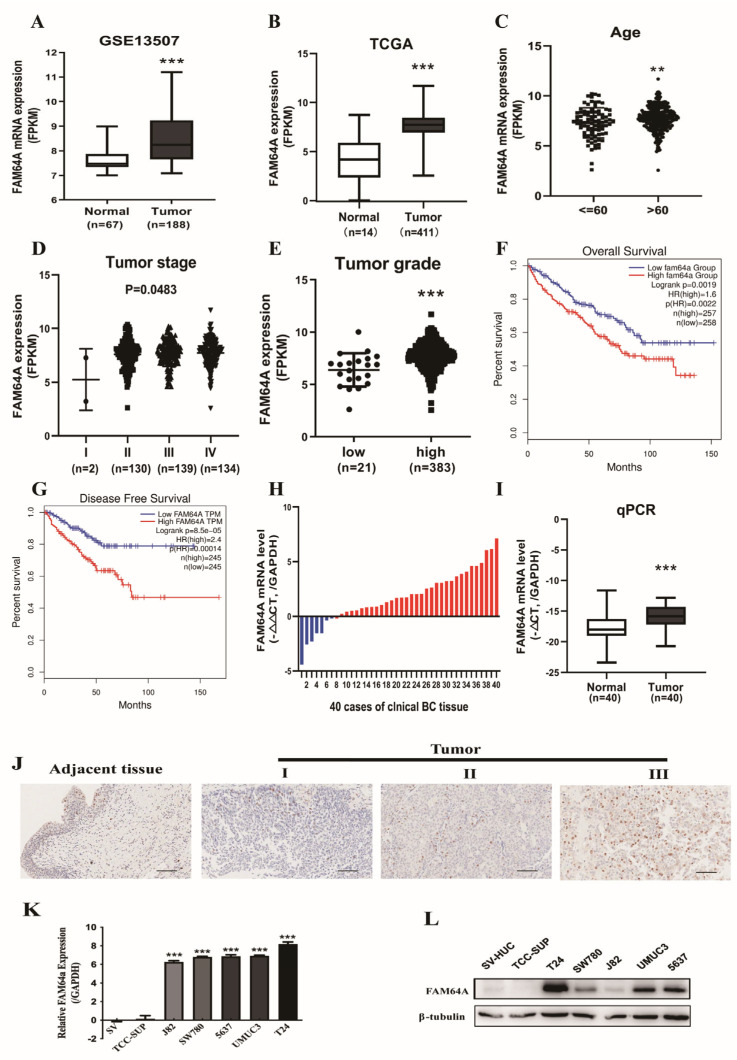
FAM64A Overexpression in Bladder Cancer Predicts Adverse Prognosis. (**A**,**B**) GSE53757 and TCGA data set showed a significantly higher expression of FAM64A in BC tissues. (**C**–**E**) FAM64A expression was positively associated with age, tumor stage and tumor grade in BC patients. (**F**,**G**) The expression of FAM64A was negatively correlated with overall survival and disease-free survival in BC patients. (**H**,**I**) The relative expression of FAM64A in 40 pairs of BC tissues and adjacent normal bladder tissues. Statistical analysis revealed that FAM64A was dramatically upregulated in 40 BC tissues compared to normal tissues. (**J**) IHC assays showed FAM64A protein expression in different tumor stages of BC tissues and adjacent normal bladder tissues. Scale bar = 50 μm. (**K**,**L**) The expression of FAM64A in BC cell lines and SV-HUC cells at both the transcriptional and translational levels. ** *p* < 0.01. *** *p* < 0.001.

Transcriptome analysis of 10 BLCA samples identified elevated FAM64A, a cell cycle regulator. Analysis of TCGA and GEO datasets confirmed significant FAM64A upregulation in BLCA tissues vs. standard controls (Figure 1A,B). High FAM64A expression correlated with advanced patient age, higher tumor stage (T-stage), and higher tumor grade (Figure 1C–E). Kaplan–Meier curves demonstrated a significant correlation between FAM64A overexpression and shorter OS, along with diminished DFS (Figure 1F,G). TCGA cohort stratification confirmed associations with clinical features (Table 1). Validation in 40 clinical BLCA samples via RT-qPCR confirmed elevated FAM64A protein and mRNA levels in tumors vs. adjacent normal tissues (Figure 1H,I). IHC results further confirmed increased FAM64A expression in bladder cancer tissues compared to adjacent non-tumorous tissues (Figure 1J, Table 1). FAM64A expression significantly correlated with pathological grade and T stage in bladder cancer patients (*p* < 0.05, Table 2). Consistent with clinical correlations, TCGA analysis demonstrated significant associations between FAM64A expression and both tumor grade and nodal stage (*p* < 0.05, Table 3). FAM64A dysregulation was evident across six BLCA cell lines (TCC-SUP, 5637, T24, UMUC3, SW780, J82), with both mRNA and protein levels—assessed by RT-qPCR and Western blot, respectively—showing marked elevation compared to the normal SV-HUC-1 reference (Figure 1K,L). Collectively, these findings demonstrate FAM64A overexpression across cellular and tissue-level bladder cancer models, correlating significantly with advancing disease severity.

### 3.2. FAM64A Knockdown Impairs Proliferative, Migratory, and Invasive Capacities in BLCA

**Figure 2:**
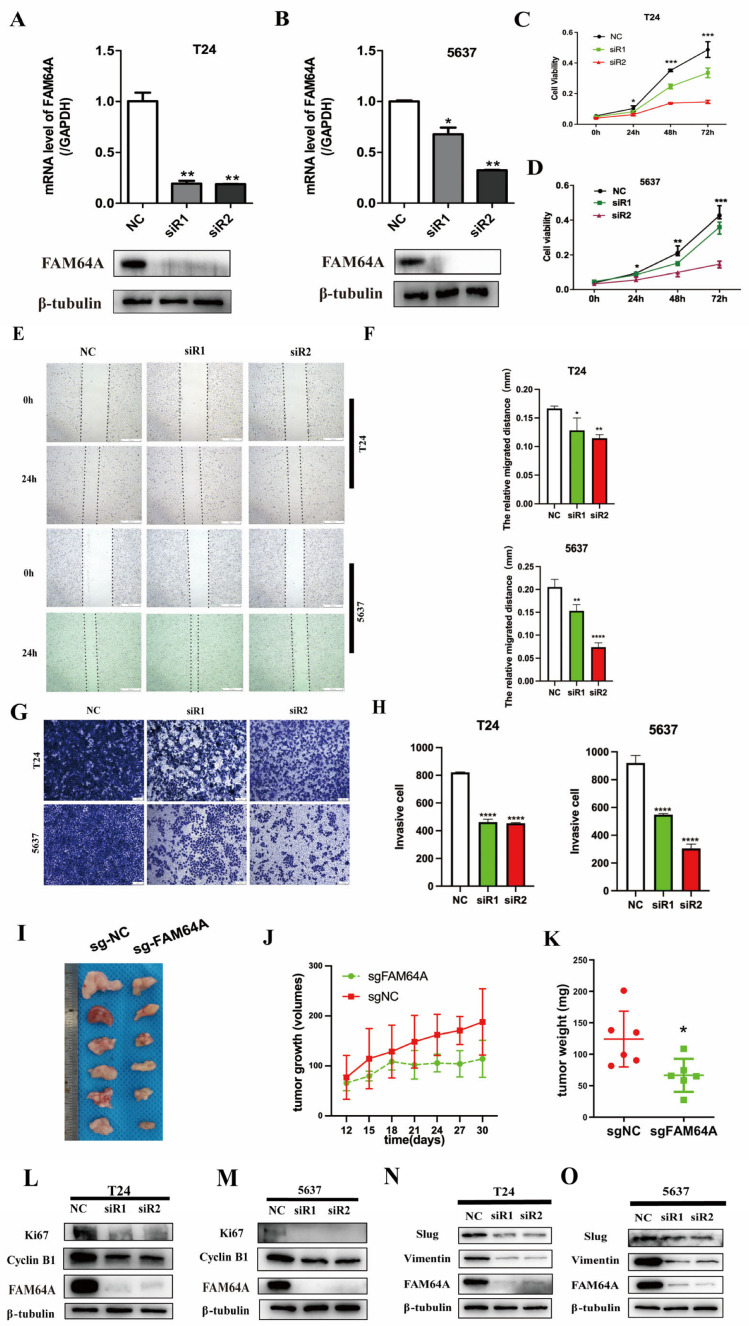
FAM64A Knockdown Impairs Proliferative, Migratory, and Invasive Capacities in BLCA. (**A**,**B**): RT-qPCR and immunoblotting verified successful FAM64A knockdown in T24 and 5637 cell lines. (**C**,**D**): CCK-8 proliferation assays demonstrated significant growth inhibition upon FAM64A silencing. (**E**–**H**): Cell motility and invasion capabilities were substantially impaired as shown by wound healing and Transwell assays. (**I**–**K**): Xenograft experiments confirmed the tumor-suppressive effect of FAM64A knockdown in vivo. (**L**,**M**): Immunoblot analysis revealed downregulation of proliferation markers Ki-67 and Cyclin B1. (**N**,**O**): EMT pathway suppression was observed following FAM64A depletion. * *p* < 0.05, ** *p* < 0.01, *** *p* < 0.001, **** *p* < 0.0001. The dotted line indicates the cell boundary.

The functional role of FAM64A in bladder carcinogenesis was investigated through siRNA-mediated knockdown in 5637 and T24 cell lines, with non-targeting siRNA (siR-NC) serving as a control. RT-qPCR and Western Blot validated successful FAM64A knockdown, evidencing significant reductions at both transcriptional and protein levels. (Figure 2A,B). FAM64A silencing markedly impaired cellular proliferation in CCK-8 assays (Figure 2C,D). Transwell invasion and wound healing assays revealed significantly reduced invasive and migratory capacities in siR-FAM64A cells (Figure 2E–H). Subcutaneous xenograft models confirmed the attenuated tumorigenicity of FAM64A-depleted cells, with significant reductions in both tumor volume and mass compared to the control group. (Figure 2J,K). Given prior reports linking FAM64A to Cyclin B1 expression and accelerated G2/M transition, we examined cell cycle regulators. Western blot confirmed that FAM64A knockdown suppressed the levels of Cyclin B1 and Ki67 (Figure 2L,M), consistent with the observed proliferation defect. FAM64A depletion significantly attenuated the expression of mesenchymal markers Vimentin and Slug, indicating suppressed EMT progression (Figure 2N,O), which aligns with the diminished invasion and migration capabilities.

Time-course analysis of Cyclin B1 expression following cell cycle synchronization indicated a delayed accumulation peak in FAM64A-knockdown cells (Figure S1A), suggesting impaired G2/M progression. Flow cytometric cell cycle analysis substantiated this finding, demonstrating that FAM64A knockdown suppressed expression of the mitotic marker MPM2 and delayed the G2 to M phase transition (Figure S1B,C).

### 3.3. FAM64A Overexpression Promotes BLCA Proliferation, Migration, and Invasion

**Figure 3:**
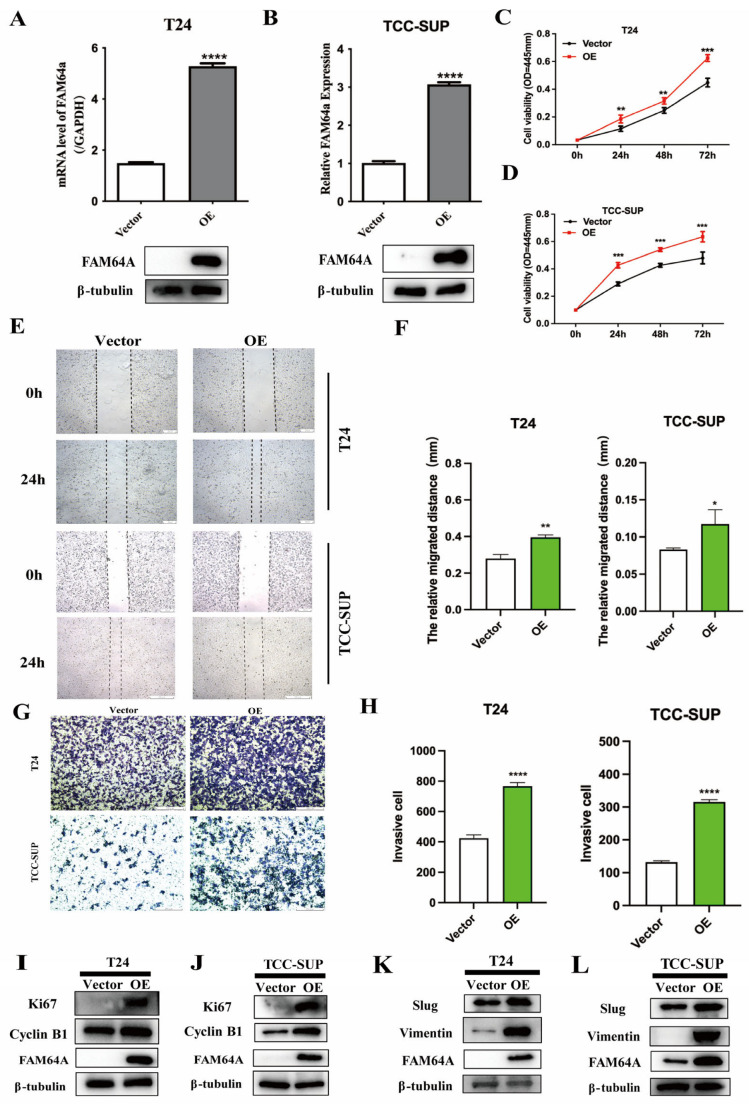
FAM64A Overexpression Promotes BLCA Proliferation, Migration, and Invasion. (**A**,**B**): Stable lentiviral transduction achieved efficient FAM64A overexpression in T24 and 5637 cells, confirmed by RT-qPCR and Western blot. (**C**,**D**): FAM64A upregulation significantly enhanced cellular proliferation in CCK-8 assays. (**E**–**H**): Overexpression of FAM64A promoted metastatic potential as evidenced by improved wound closure and Transwell invasion. (**I**,**J**): Immunoblot analysis showed upregulation of Ki-67 and Cyclin B1 in FAM64A-overexpressing groups. (**K**,**L**): Activation of the EMT program was confirmed in cells with elevated FAM64A expression. * *p* < 0.05, ** *p* < 0.01, *** *p* < 0.001, **** *p* < 0.0001. The dotted line indicates the cell boundary.

Similarly, T24 and TCC-SUP cell lines were transduced with lentiviral vectors encoding FAM64A (OE-FAM64A) or a negative control vector (LV238). Experimental validation documented substantial upregulation of FAM64A, with both qPCR and immunoblotting analyses confirming enhanced expression across nucleic acid and protein domains (Figure 3A,B). Functional assessment of FAM64A-overexpressing bladder cancer cells was performed using CCK-8 proliferation assays, Transwell invasion analysis, and wound healing migration assays to quantify oncogenic phenotypes. CCK-8 results revealed a significant potentiation of proliferative capacity in both 5637 and T24 cells upon FAM64A overexpression (Figure 3C,D). Transwell invasion and wound healing assays consistently demonstrated a markedly enhanced invasive and migratory capability in these cell lines following FAM64A upregulation (Figure 3E–H). Corroborating its pro-proliferative role, Western blotting detected concurrent increases in Cyclin B1 levels and the proliferation marker Ki-67 in FAM64A-overexpressing cells (Figure 3I,J). Furthermore, FAM64A upregulation concomitantly elevated the mesenchymal markers Vimentin and Slug, indicating activation of the EMT pathway (Figure 3K,L), aligning with the observed enhancement in cellular invasion and migration. To investigate the cell cycle dynamics, Cyclin B1 expression was monitored at distinct time points. The analysis demonstrated an accelerated accumulation of Cyclin B1 protein in cells overexpressing FAM64A compared to controls (Figure S1D). This temporal shift suggests that FAM64A accelerates the traversal of the G2/M checkpoint.

### 3.4. FAM64A Activates PI3K/mTORC2/AKT Signaling to Mediate EMT

**Figure 4:**
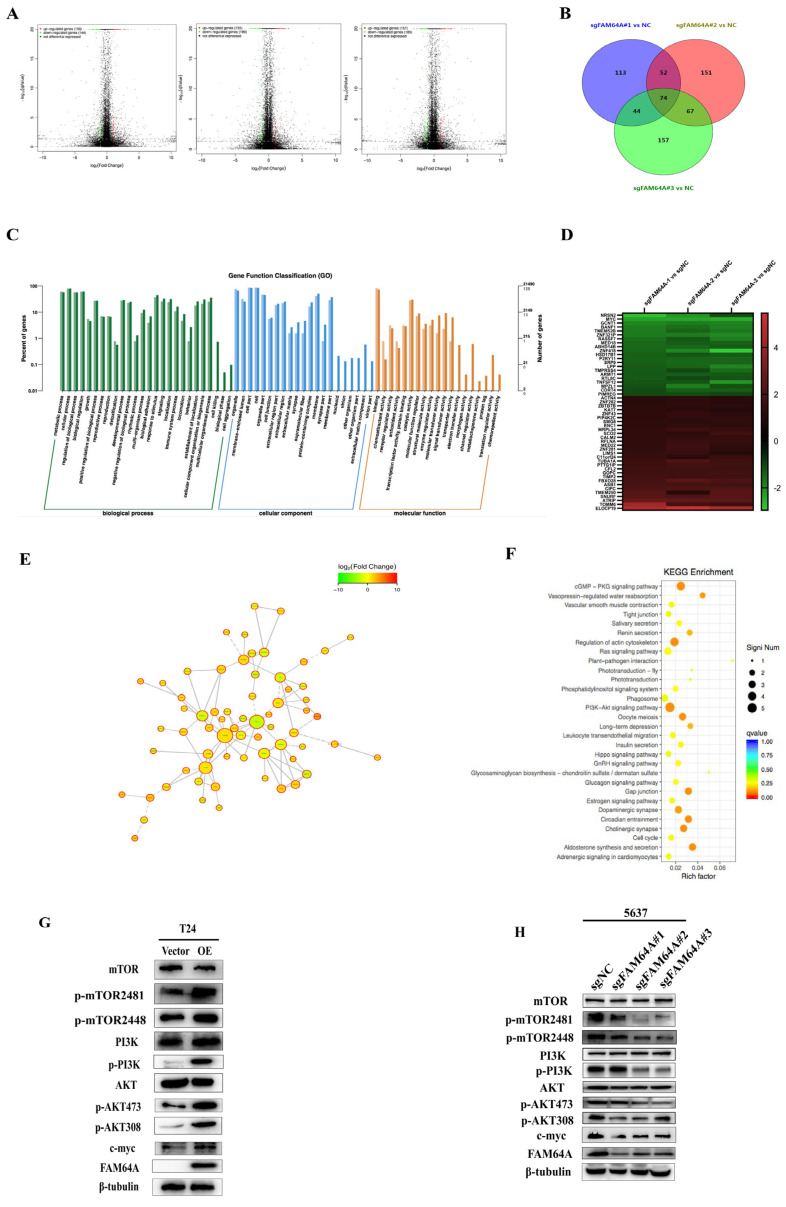
FAM64A Triggers PI3K/mTORC2/AKT Signaling. (**A**) Bioinformatics analysis using GEO2R identified differentially expressed genes modulated by FAM64A in BLCA. (**B**) Intersection analysis through Venn diagrams yielded 74 consensus targets. (**C**) Gene Ontology assessment characterized functional attributes of the integrated DEGs. (**D**) Cluster heatmap organized the top 50 most significant DEGs from consolidated microarray data. (**E**) PPI network construction pinpointed pivotal nodes within the FAM64A—correlated transcriptome. (**F**) KEGG analysis uncovered pathway enrichments among the selected candidates. (**G**) Immunoblotting confirmed PI3K/mTORC2/AKT pathway activation following FAM64A overexpression. (**H**) FAM64A silencing attenuated PI3K/mTORC2/AKT signaling in Western blot assays.

Transcriptome sequencing identified 283 DEGs (139 up, 144 down; *p* < 0.05, |logFC| ≥ 2) in sgFAM64A vs. sgNC cells (Figure 4A). Venn analysis identified 74 overlapping genes (Figure 4B). GO enrichment revealed key biological functions (Figure 4C). Heatmap visualization highlighted the top 50 DEGs (Figure 4D). STRING network analysis and KEGG functional annotation pinpointed the PI3K/mTORC2/AKT pathway as significantly enriched (Figure 4E,F).

**Figure 5:**
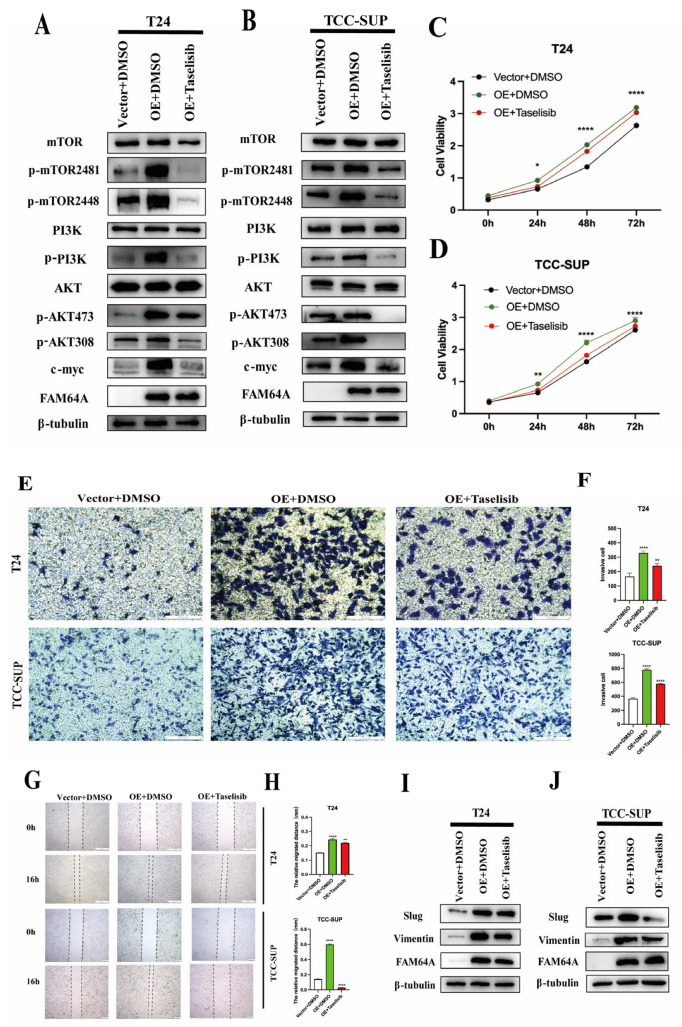
FAM64A Promotes EMT via PI3K/AKT Signaling and Is Reversed by PI3K Inhibition. (**A**,**B**): The PI3K inhibitor Taselisib abrogated FAM64A-driven PI3K/mTORC2/AKT pathway activation in immunoblot assays. (**C**,**D**): Cellular proliferation potentiated by FAM64A overexpression was neutralized by Taselisib treatment in CCK-8 assays. (**E**–**H**): Taselisib eliminated the promotive effects of FAM64A on cell motility and invasion in wound healing and Transwell assays. (**I**,**J**): EMT program activation induced by FAM64A was effectively reversed following Taselisib administration. * *p* < 0.05, ** *p* < 0.01, **** *p* < 0.0001. The dotted line indicates the cell boundary.

Catalytic activation of membrane-recruited PI3K converts PIP_2_ into the key second messenger PIP_3_ through phosphorylation at the 3-OH position of the inositol ring [36]. PIP_3_ acts as an essential membrane-docking platform that recruits Akt (Protein Kinase B) via high-affinity interactions with its pleckstrin homology (PH) domain. This interaction drives Akt translocation from the cytosol to the inner plasma membrane, inducing a conformational change that positions Akt for phosphorylation-mediated activation [37,38]. Subsequently, PI3K-associated kinases, primarily PDK1, phosphorylate and activate Akt [39,40]. Activated Akt regulates core cellular functions, including proliferation, cell cycle progression, apoptosis, autophagy, and survival, through the phosphorylation of downstream targets. To delineate the functional relationship between FAM64A expression and the PI3K/AKT/mTOR signaling pathway, we performed validation at the protein level. Western blot analysis revealed that FAM64A overexpression activated the phosphorylation (activation) status of key components within the PI3K/mTORC2/AKT pathway (Figure 4G). Conversely, FAM64A knockdown suppressed this signaling cascade (Figure 4H). To further establish causality, we utilized the PI3K pathway inhibitor Taselisib in FAM64A-overexpressing T24 and TCC-SUP cell lines. Protein-level analysis confirmed that Taselisib treatment effectively inhibited PI3K/mTORC2/AKT signaling activation induced by FAM64A overexpression (Figure 5A,B). Concurrently, to assess the functional consequences of PI3K inhibition, we conducted CCK-8 proliferation assays, Transwell invasion assays, and wound healing migration assays. Taselisib treatment potently inhibited proliferative, invasive, and migratory capacities in bladder cancer cells (Figure 5C–H). Furthermore, we examined the status of the epithelial–mesenchymal transition (EMT) pathway following PI3K inhibition. Taselisib abrogated PI3K/mTORC2/AKT signaling, resulting in significant EMT marker suppression (Vimentin, Slug) (Figure 5I,J).

**Figure 6:**
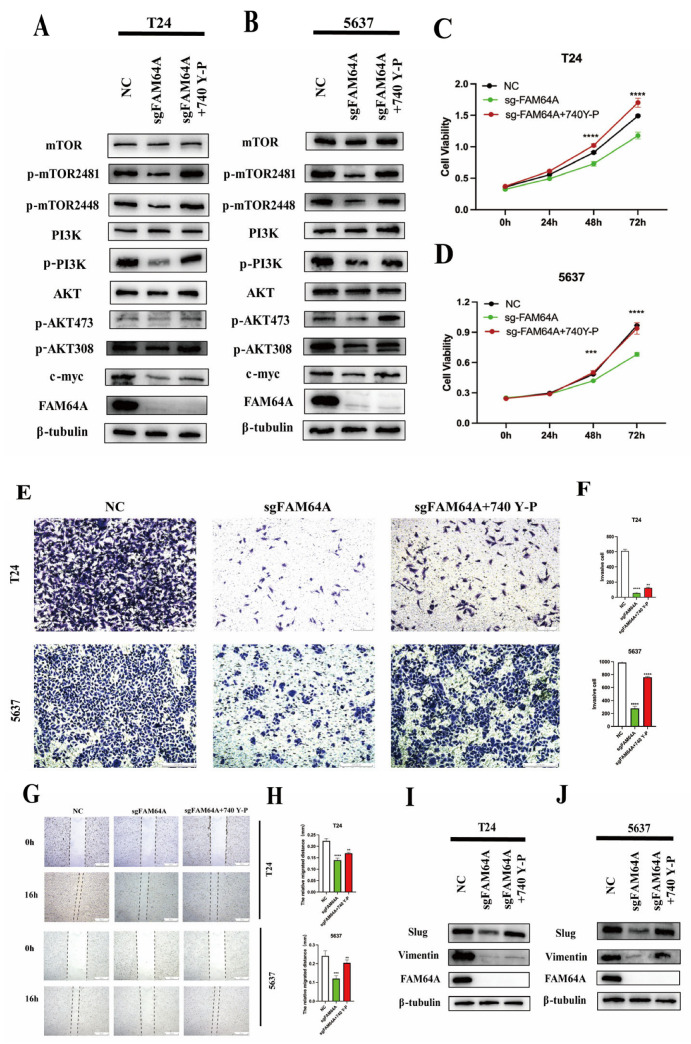
PI3K agonist 740Y-P counteracts the inhibitory effects of FAM64A deficiency on EMT. (**A**,**B**): The PI3K/mTORC2/AKT pathway suppression following FAM64A downregulation was alleviated by 740Y-P treatment in immunoblot assays. (**C**,**D**): Reduced proliferative activity due to FAM64A knockdown was recovered upon 740Y-P administration in CCK-8 experiments. (**E**–**H**): 740Y-P compensated for the diminished migratory and invasive capacities associated with FAM64A loss in wound-closure and Transwell invasion assays. (**I**,**J**): Expression levels of key EMT markers were re-established in FAM64A-suppressed cells following 740Y-P exposure. ** *p* < 0.01, *** *p* < 0.001, **** *p* < 0.0001. The dotted line indicates the cell boundary.

Subsequently, the essential role of the PI3K signaling pathway within the FAM64A regulatory network was confirmed through rescue experiments. Treatment with the PI3K activator 740Y-P effectively reversed the suppression of PI3K/mTOR/AKT signaling caused by FAM64A knockdown (Figure 6A,B). Additional functional tests—including CCK-8 proliferation, Transwell invasion, and wound-healing migration assays—demonstrated that 740Y-P significantly restored the reduced proliferative, invasive, and migratory abilities resulting from FAM64A downregulation (Figure 6C–H). Furthermore, reactivating PI3K signaling led to increased levels of epithelial–mesenchymal transition (EMT) markers Vimentin and Slug, confirming the pathway’s key role in FAM64A-driven EMT progression (Figure 6I,J).

Collectively, these data establish FAM64A as an EMT driver in bladder cancer through the activation of the PI3K/mTORC2/AKT pathway, propelling tumor progression and metastatic dissemination.

## 4. Discussion

Bladder cancer (BLCA) ranks among the most common genitourinary malignancies globally. Its pronounced propensity for metastasis and recurrence poses significant challenges for clinical management, underscoring the critical importance of identifying novel molecular targets for this disease. Recent advancements in high-throughput sequencing have substantially enhanced our understanding of cancer biology. In our preliminary research, we first report the upregulation of FAM64A, a gene implicated in cell cycle regulation, in BLCA through transcriptomics: tumor tissues showed significantly higher expression than paired standard controls. A literature review indicates that FAM64A is a cell cycle-associated gene; prior studies have confirmed its elevated expression across various malignancies, including breast, prostate, and gastric carcinomas, where it promotes tumorigenesis. However, its mechanistic contributions to bladder carcinogenesis and disease advancement remain poorly characterized. Our study provides the first evidence establishing FAM64A as an oncogenic factor in bladder cancer.

Interrogation of public transcriptomic datasets (TCGA and GEO) revealed marked transcriptional upregulation of FAM64A in BLCA, which was significantly associated with adverse clinical outcomes. Higher tumor grade and worse survival outcomes were associated with progressively elevated FAM64A expression levels. Subsequent validation using clinical BLCA patient tissues and cell lines, at both the RNA and protein levels, corroborated these findings. FAM64A mRNA and protein expression were consistently higher in tumor tissues and cells relative to matched adjacent normal tissues.

Functionally, FAM64A knockdown suppressed the growth, invasion, and migration of BLCA cells. Conversely, FAM64A amplification exacerbated malignant behaviors. In vivo validation demonstrated that FAM64A knockdown suppressed tumorigenicity, resulting in a significant decrease in both tumor volume and mass.

Epithelial–mesenchymal transition (EMT) refers to a reprogramming process in which epithelial cells lose their adhesive properties and acquire migratory mesenchymal characteristics [41,42]. Compelling evidence positions EMT as a critical mediator of malignancy invasion and metastatic dissemination [43,44], including a demonstrated association with bladder cancer invasion, facilitating tumor cell dissemination and metastasis [31,45]. Our results demonstrate that FAM64A promotes the growth, invasion, migration, and cell cycle progression of BLCA cells, indicating its role as a key regulator of EMT in bladder cancer cells. Western blot analysis confirmed increased expression of established EMT molecular markers, Vimentin and Slug, in BLCA cells. Furthermore, prior studies have shown that FAM64A promotes EMT in breast cancer cells [46]. FAM64A (RCS1/PIMREG/CATS) is a cell cycle-related gene critically involved in cellular proliferation. It exerts significant biological functions across various cell types by accelerating cell cycle progression [47].

The cell cycle represents the essential biological process that regulates cellular duplication and growth [48]. Precise regulation of this cycle is essential for maintaining genomic stability, integrity, and normal cellular function. It comprises four distinct phases: G0/G1, S, G2, and M [49]. Cell cycle progression, whether activation or inhibition, is controlled at critical regulatory checkpoints, including the G1/S transition, the G2/M boundary, and the metaphase-to-anaphase transition. Dysregulation at these checkpoints is a frequent driver of tumorigenesis [50,51]. For instance, the G2/M checkpoint, governed by the CDK1/Cyclin B complex, prevents cells with DNA damage from entering mitosis [52]. Our results demonstrate that FAM64A knockdown reduces the levels of Cyclin B1 and Ki-67, while FAM64A overexpression enhances their expression. Cell cycle synchronization followed by time-course protein analysis revealed that FAM64A knockdown delays the peak expression of Cyclin B1. Flow cytometry cell cycle analysis further confirmed that FAM64A knockdown delays the peak accumulation of cells in G2/M phase, whereas its overexpression advances this peak.

To elucidate the underlying molecular mechanism, we employed transcriptome sequencing. Utilizing GEO2R, we identified differentially expressed genes (DEGs) regulated by FAM64A in BLCA cells. A systematic screening of biological functions was conducted through GO term and KEGG pathway analyses to map the comprehensive functional landscape. KEGG analyses revealed PI3K-AKT cascade activation as a top FAM64A-associated pathway, positioning it upstream of EMT in bladder carcinogenesis.

The PI3K/mTORC2/AKT axis constitutes a central signaling hub orchestrating essential cellular processes. This pathway orchestrates diverse cellular functions, notably cellular growth, proliferation [53], apoptotic regulation [54,55], and metabolic control [56]. Mechanistic studies have established that dysregulated pathway activity contributes to the pathogenesis of a diverse array of conditions, including cancer [36,57], diabetes [58], hematological disorders [59], and cardiovascular diseases [60,61]. Collectively, our findings define FAM64A as an upstream component in the hierarchical signaling architecture that regulates PI3K/mTORC2/AKT activation. Significantly, pharmacological blockade of this pathway using Taselisib attenuated malignant phenotypes—proliferation, migration, and invasion—in BLCA cells while concomitantly downregulating EMT markers. In contrast, treatment with the PI3K activator 740 Y-P yielded opposing results. This indicates that FAM64A promotes the expression of EMT-related markers Snail2 (Slug) and Vimentin by activating the PI3K/mTORC2/AKT signaling pathway. Snail2, acting as a transcription factor, induces the initiation of epithelial–mesenchymal transition. Concurrently, Vimentin facilitates EMT by altering cell morphology and motility, thereby collectively promoting the initiation and metastasis of bladder cancer [29]. These data confirm that FAM64A drives EMT-mediated progression in bladder cancer predominantly through PI3K/mTORC2/AKT pathway activation. These findings collectively suggest that FAM64A could serve as a prognostic biomarker detectable via tissue biopsy and represents a potential therapeutic target for bladder cancer. However, it is important to acknowledge the limitations of our current study. The precise molecular mechanism by which FAM64A activates the PI3K/mTORC2/AKT pathway remains unclear. Elucidating this specific mechanism will be a critical focus of our future research.

## 5. Conclusions

This investigation establishes FAM64A as a progression-linked oncogene in bladder cancer, exhibiting upregulated expression in BLCA tissues and cellular models that escalates with tumor advancement. Our results delineate FAM64A as a molecular determinant of bladder cancer progression, where it accelerates cell cycle transitions and initiates EMT through PI3K/mTORC2/AKT signaling. This pathway activation culminates in enhanced tumor growth and metastasis, revealing FAM64A as a therapeutic vulnerability and clinically relevant prognostic indicator.

## Figures and Tables

**Table 1:** Expression levels of FAM64A in normal tissues and bladder cancer (IHC).

Tissue Type	Expression of FAM64A		No. of Case (n)	χ^2^	*p*
	Low	High			
Normal	6	1	7	5.381	0.02036 *
Carcinoma	20	31	51		
Total	26	32	58		

*T*-test, * *p* < 0.05.

**Table 2:** Correlation between FAM64A expression and the clinico-pathologic features of patients with bladder cancer (IHC).

Clinico-Pathologic Feature	Expression of FAM64A		No. of Case (*n* = 51)	χ^2^	*p*
	Low	High			
Gender					
male	19	25	44	2.115	0.14585
female	1	6	7		
Age					
≤60	4	8	12	0.228	0.63301
>60	16	23	39		
Survive (years)					
≤5	16	23	39	0.228	0.63301
>5	4	8	12		
Pathological type					
I	2	0	2	6.263	0.04365 *
II	17	23	40		
III	1	8	9		
Primary tumor stage					
T1	9	11	20	5.088	0.02409 *
T2–T4	14	37	31		

χ^2^, * *p* < 0.05.

**Table 3:** Correlation between FAM64A expression and the clinico-pathologic features of patients with bladder cancer (TCGA).

Clinico-Pathologic Feature	Expression of FAM64A		No. of Case	χ^2^	*p*
	Low	High			
Gender					
male	141	170	311	3.297	0.0010
female	62	34	96		
Age					
≤60	54	160	214	10.51	0.0001
>60	150	44	194		
Survive (years)					
≤5	179	181	360	0.1730	0.8626
>5	24	23	47		
Tumor Grade					
High	184	199	383	3.362	0.0008
Low	18	3	21		
Pathological type					
I	2	0	2	1.471	0.2253
II	68	62	130		
III	70	69	139		
IV	62	72	134		
T stage					
T0–1	2	2	4	3.356	0.3399
T2	59	60	110		
T3	90	103	193		
T4	35	23	58		
Lymph note					
N0	128	108	236	4.442	0.0351 *
N1	19	27	46		
Nx	14	22	36		
Metastasis					
M0	107	89	196	3.483	0.0620
M1	6	5	11		
Mx	89	108	197		

χ^2^, * *p* < 0.05.

## Data Availability

The original data of the study, including gels and blots, can be found in the article/Supplemental File, and further inquiries can be directed to the corresponding authors.

## Supplementary Materials

The following supporting information can be downloaded at: https://www.mdpi.com/article/10.3390/cancers18030540/s1, Figure S1: FAM64A expression can affect the transition from the G2 phase to the M phase.

## Abbreviations

The following abbreviations are used in this manuscript:

BLCA	Bladder cancer
MIBC	Muscle-invasive Bladder cancer
NMIBC	Non-muscle-invasive Bladder cancer
IHC	Immunohistochemistry
