# Supplementary figures and images for: FAM64A Potentiates Bladder Carcinoma Tumorigenesis and Metastasis Through PI3K/mTORC2/AKT Pathway Activation
Source: Cancers (Basel). 2026 Feb 6;18(3):540. doi: 10.3390/cancers18030540 (PMC12896460; doi:10.3390/cancers18030540)

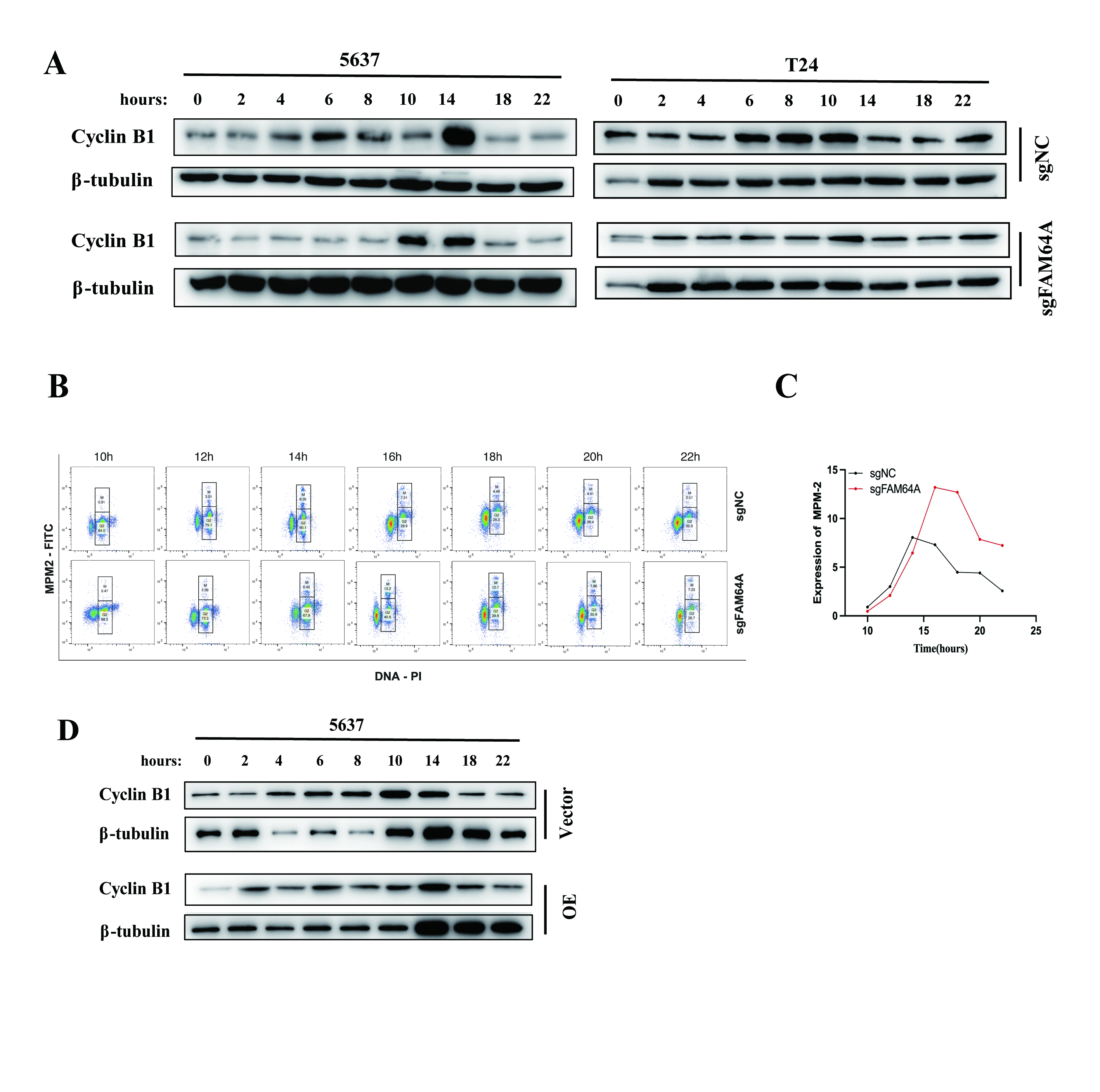

Supplement: Supplementary file 1 [file cancers-18-00540-s001.zip › cancers-4105725-supplementary.tif]
